# Wearable Sensors and Artificial Intelligence for Ecological Knee Osteoarthritis Assessment: Development and Feasibility of a Hybrid Digital Phenotyping Framework

**DOI:** 10.3390/s26113563

**Published:** 2026-06-03

**Authors:** Jean Mapinduzi, Kim Daniels, Oyéné Kossi, Jonas Verbrugghe, Bruno Bonnechère

**Affiliations:** 1 REVAL Rehabilitation Research Center, Faculty of Rehabilitation Sciences, Hasselt University, 3590 Diepenbeek, Belgium; jean.mapinduzi@uhasselt.be (J.M.); kim.daniels@pxl.be (K.D.); oyene.kossi@uhasselt.be (O.K.); jonas.verbrugghe@uhasselt.be (J.V.); 2 Technology-Supported and Data-Driven Rehabilitation, Data Sciences Institute, Hasselt University, 3590 Diepenbeek, Belgium; 3 TechnoRehab Lab^2^, Filière de Kinésithérapie et de Réadaptation, Département des Sciences Cliniques, Institut National de Santé Publique (INSP), Bujumbura 6807, Burundi; 4 Department of PXL—Healthcare, PXL University of Applied Sciences and Arts, 3500 Hasselt, Belgium; 5 ENATSE, National School of Public Health and Epidemiology, Université de Parakou, Parakou P.O. Box 123, Benin

**Keywords:** osteoarthritis, smart healthcare, AI-enabled sensing, digital phenotyping, wearable sensors, remote patient monitoring, precision diagnosis

## Abstract

Osteoarthritis (OA) is a highly prevalent musculoskeletal disorder and a major cause of disability, posing growing challenges for healthcare systems worldwide. Conventional supervised clinical assessments provide valuable insights but are largely limited to cross-sectional snapshots and often fail to reflect the variability of real-world functioning, physical activity patterns, and symptom fluctuations experienced by individuals with OA, especially those with knee OA. This perspective introduces a multisensor digital phenotyping framework for smart knee OA assessment, integrating supervised laboratory evaluations with unsupervised continuous monitoring in daily living environments using wearable sensors, smart insoles, activity trackers, and mobile devices. Feasibility was tested in 40 participants (20 knee OA patients, 20 controls). Raw data from questionnaires, electronic goniometry, dynamometry, force plate, connected insoles, and seven-day home monitoring were harmonized via a standardized pipeline aligned with the ICF framework. The pipeline employed anomaly detection, missing data imputation, z-score normalization, and cloud-based storage. This framework is envisioned to facilitate advanced data integration and machine-learning-ready analytics, enabling longitudinal monitoring, pattern recognition, and individualized health profiling. By conceptually bridging cross-sectional and continuous sensing modalities, this approach has the potential to enhance ecological validity, support earlier identification of functional decline, and inform data-driven clinical decision-making. Key methodological, technological, and ethical challenges—including data quality, interpretability, privacy, digital literacy, and clinical adoption—are also highlighted. Overall, this paper underscores the promise of AI-enabled multisensor digital phenotyping to advance smart, personalized, and precision healthcare for individuals with knee OA.

## 1. Context

Osteoarthritis (OA) is a leading cause of disability and a significant contributor to societal costs among older adults [1]. Its prevalence increases consistently with age, affecting approximately 20% of individuals aged 60 years or older, with a notably higher incidence among women [2,3]. This rising prevalence is attributed to several factors, including aging, obesity associated with physical inactivity, prior trauma, sedentary lifestyles, and occupations [1,3]. OA is characterized by structural and functional deterioration of the articular and periarticular elements. Clinically, it manifests as chronic pain, reduced range of motion (ROM) in the affected joint, diminished proprioception, weakened surrounding musculature, audible crepitus, and occasional joint effusion [4,5]. This clinical presentation significantly impacts the daily functioning and participation of individuals, including their ability to perform activities of daily living, maintain employment, and engage in social interactions [5,6].

Although OA can affect all joints of the body, the knee, as one of the primary weight-bearing joints, is the most commonly affected by OA and is the major contributor to increased walking-related fatigue and disability in older adults [7]. Many individuals report moderate to severe pain and functional limitations, often necessitating joint replacement [2,3].

As the global population continues to age, addressing these clinical symptoms has become increasingly important, necessitating effective strategies for the treatment and management of OA [8]. To optimize care, healthcare professionals must perform comprehensive and objective evaluations to inform individualized clinical decisions and prognostications [9].

Traditionally, patient assessments have been conducted in clinical or laboratory settings under the supervision of qualified healthcare professionals. These supervised assessments, whether qualitative or quantitative, provide a single snapshot of patient outcomes, and are influenced by factors such as the Hawthorne effect, timing of measurements, and the presence of healthcare personnel [10]. Patients often perform better when they are aware of being observed [9,10,11,12,13]. Furthermore, supervised assessments are limited in their ability to capture diverse clinically relevant events, such as prolonged physical activity, fatigue interference, or functioning during pain flare-ups, which are often episodic or fluctuate in complex patterns [9]. Conducting these assessments also requires significant time and trained personnel, which may not always be available. To reliably and objectively capture real-time data, it is essential to assess patients over extended periods in their daily living environments without supervision [9].

Recent advances in wearable sensing technologies, mobile health platforms, and artificial intelligence (AI) offer unprecedented opportunities to transition from episodic clinical assessments toward continuous, data-driven, and ecologically valid monitoring of patients’ functional status. Within this context, integrating intelligent sensing systems and digital phenotyping approaches into OA care has the potential to support precision diagnosis, personalized rehabilitation, and smarter healthcare delivery.

Unsupervised assessments, which involve the continuous quantitative evaluation of mobility in home and daily living environments using mobile health technologies, offer several advantages [9]. First, they are timely and cost-effective, allowing data to be collected independently of healthcare professionals’ availability. Second, they empower patients by enabling active participation in their care [10,14,15]. Using personal devices such as smartphones, smartwatches, and wearable sensors, patients can collect clinical information in real-world settings and receive feedback on their daily living performance [14,15]. These devices can measure parameters such as step count, physical activity duration, sedentary behavior (i.e., inactivity alerts), heart rate, and distance walked, providing valuable insights into physical activity levels. Compared to traditional clinical assessments, unsupervised evaluations offer unparalleled accessibility and flexibility. Additionally, integrating the combination of supervised and unsupervised assessment methods into clinical practice has the potential to identify key factors influencing physical activity behaviors. In this perspective, we propose a holistic approach that integrates various evaluation modes (supervised and unsupervised) and timeframes (cross-sectional and continuous data collection). We aim to provide recommendations for incorporating unsupervised clinical assessments into routine care and future research, thereby advancing the understanding and management of knee OA.

## 2. AI-Enabled Digital Phenotyping and Intelligent Sensing for Personalized Knee OA Care

Capturing diverse clinically relevant events is challenging during cross-sectional evaluation in laboratory or hospital settings, only providing a unidimensional snapshot of the patients. Such events often unfold over extended periods (i.e., total physical activity levels), occur infrequently (i.e., falls or freezing episodes, activity-related fatigue), take place during nocturnal hours (i.e., sleep disturbances), or exhibit complex and fluctuating patterns (i.e., responses to medication intake) [9]. These limitations hinder the holistic assessment of individuals with OA in general, especially those with knee OA, emphasizing the need for innovative methodologies. Digital technology presents a promising opportunity to revolutionize current assessment approaches [15,16,17].

DP, defined as the moment-by-moment collection and analysis of data from digital devices, serves as a valuable complement to traditional methods of disease prevention, assessment, prognostication, treatment, and management [9,18]. This innovative approach enables new ways to measure disease progression and therapeutic responses in ways that are meaningful to patients [16,17,18,19]. It holds particular significance for conditions like knee OA, which are characterized by functional impairments and activity limitations, and where tracking the evolution of the patients through the course of the disease is of the utmost importance [18]. By offering ecologically valid and dynamic assessments, digital phenotyping has the potential to transform our understanding of the complexities underlying impairments, activity limitations, and their impact on daily life [20].

Unlike traditional clinical assessments that rely heavily on subjective observations and self-reported data, DP leverages digital technologies to gather objective, real-time data on individuals’ physical activity patterns, physiological responses, and behaviors within their natural environments [20]. This approach enhances the accuracy and relevance of assessments, capturing aspects of patient functioning that would otherwise remain unobserved in clinical settings.

Several online platforms and digital tools (e.g., wearable devices, mobile health applications, remote monitoring systems, artificial intelligence and machine learning) have been developed to enable continuous health monitoring, empowering individuals to track their health status over time [18,21]. The advancements in telemedicine and the transition to a home-based DP framework allow clinicians and researchers to overcome many traditional assessment limitations. This paradigm shift provides a more holistic and ecologically valid understanding of OA patient functioning [21].

By integrating DP with conventional approaches, clinicians can gain valuable insights into the daily experiences and functional challenges of knee OA patients. Such insights can inform clinical decision-making and guide the development of tailored interventions aimed at improving patients’ quality of life [20]. Continuous monitoring through wearable devices, smartphone applications, and other digital tools enables clinicians to analyze pathological patterns in real time, identify key areas of concern, and implement targeted interventions to address these issues effectively [20]. This method allows for more personalized and precise assessments, adapting to the specific needs and preferences of each patient. Ultimately, it contributes to enhancing functional autonomy, well-being, and participation in daily activities for individuals with knee OA [22].

This novel approach represents a significant step forward in the assessment and management of knee OA. It highlights the potential of digital wearable technologies to bridge gaps in current methodologies, offering a deeper and more comprehensive evaluation of the condition and its impact on patients’ lives.

## 3. Data Collection Pipeline

Data collection is conducted based on the International Classification of Functioning, Disability and Health (ICF) framework aligning with the principles of the biopsychosocial model. Applying this model to knee OA facilitates the identification of impairments, activity limitations, and the associated impact on participants’ quality of life [23,24].

To comprehensively evaluate impairments and physical functioning in patients with knee OA, we are developing a comprehensive multidimensional data collection approach. This approach utilizes a combination of supervised assessments (conducted in standardized settings such as laboratories, hospitals, or rehabilitation centers) and unsupervised assessments (performed in participants’ natural ecological environments). Data are gathered using both cross-sectional and continuous collection modes. This approach enables a more holistic understanding of participants’ functional status and day-to-day experiences.

Data collection incorporates various technologies used in rehabilitation, including wearable activity trackers and mobile applications [24,25,26,27,28,29]. These tools enable the continuous capture of diverse data, including physiological metrics, physical functioning, and physical activity levels. In addition to this prospective data collection approach, a hybrid cross-sectional method is employed, blending traditional and innovative techniques to provide a comprehensive dataset.

On the one hand, traditional methods include structured interviews for collecting demographic data and self-administered questionnaires to gather subjective information on physical functioning, levels of kinesiophobia, physical activity profiles, and quality of life.

On the other hand, innovative methods involve the cross-sectional collection of objective data using new technologies [17,30,31,32]. These include measurements of range of motion, muscle strength, bipedal balance, and gait analysis captured with modern assessment tools and technologies.

Table 1 provides detailed information on the specific tools and techniques utilized for each data collection component as well as their psychometric properties.

## 4. From Supervised to Unsupervised Assessment

There is a clear and growing trend toward progressively shifting from standalone traditional supervised assessments to a combination of supervised and unsupervised methodologies, driven by advancements in digital health technologies and the need for more ecologically valid and patient-centered approaches [9]. In this section, we present the progressive steps of our multidimensional data collection framework, transitioning from conventional supervised clinical assessments to advanced DP methodologies. The main goal of integrating DP methodologies is to improve the quality of clinical assessment by complementing the traditional approach rather than replacing it, ensuring good insights into clinical decision-making. The complete list and description of the text is presented in Supplementary Material S1 and summarized in Table 1.

In summary, the framework begins with supervised clinical assessments using validated questionnaires and tests. Questionnaires include: Visual Analog Scale (VAS) for pain intensity; Western Ontario and McMaster Universities Osteoarthritis (WOMAC) for physical functioning (pain, stiffness, function); Tampa Scale for kinesiophobia; Short form-20 (SF-20) for quality of life; and Global Physical Activity Questionnaire (GPAQ) for physical activity levels. Clinical tests comprise the 6 min walk test (6-MWT) for activity limitation and the Five-Times Sit-to-Stand Test (5xSTS) for lower body strength, mobility, and balance.

The cross-sectional phase incorporates objective technology-based measurements: an electronic goniometer for joint range of motion, an electronic dynamometer for isometric muscle strength, a force plate for balance parameters (following mCTSIB protocol), and connected insoles paired with the 6-MWT for detailed gait analysis (cadence, stride length, asymmetry, fatigability).

The framework then transitions to unsupervised continuous monitoring in natural environments for over one week. A smartwatch (Polar M200) tracks physical activity levels. The wear time rules were: minimum valid day = ≥10 h of wear during waking hours (6:00–24:00). Minimum valid week = ≥4 valid days including ≥1 weekend day. Non-wear was defined as ≥ 60 consecutive minutes of zero counts.

The final stage integrates digital phenotyping methodologies to enable personalized, adaptive care.

## 5. Data Synergy: Organize, Analyze and Optimize

The multisensor data architecture described in this framework is intentionally designed to be compatible with artificial intelligence and machine learning workflows. By harmonizing heterogeneous data streams from wearable sensors, clinical assessments, and mobile devices, the platform enables scalable feature extraction, pattern recognition, and predictive modeling, which are essential components of AI-driven smart healthcare systems.

**Figure 1:**
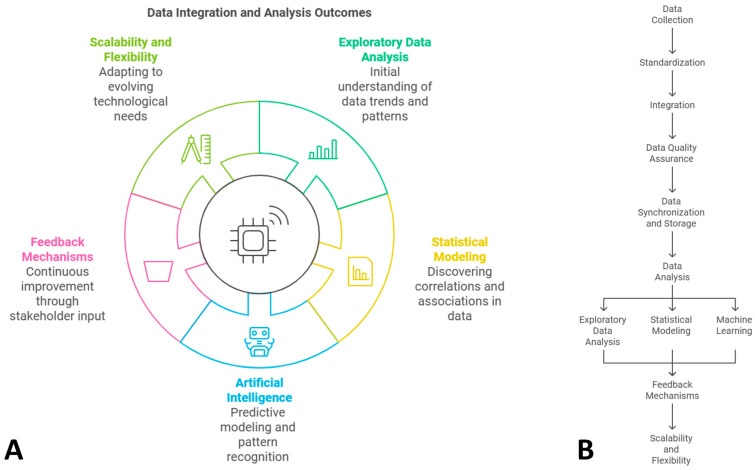
(**A**) Illustrative overview of data integration and analysis. (**B**) Flow of data collection and analysis.

Building on the data collection methods outlined above, it is essential to establish a robust methodology for data storage, synchronization, and analysis (see Figure 1 for an overview of the different steps). To address these requirements, we have implemented a state-of-the-art data integration platform designed to ensure compatibility across diverse device platforms and data formats.

The platform incorporates advanced algorithms for anomaly detection and correction, minimizing data errors and enhancing the reliability of multisource data aggregation. This functionality is critical for aligning various technological inputs with their corresponding clinical applications, enabling seamless integration of data from wearable devices, sensors, and digital applications.

Developing such an integrated platform is vital for fully leveraging the potential of the collected data. By facilitating the synthesis of multisource inputs, the platform enables researchers and clinicians to conduct comprehensive analyses that go beyond individual assessments. Without this integration, analyses would be limited to isolated evaluations, restricting opportunities to uncover complex interrelationships and generate deeper insights into the progression, management, and rehabilitation of knee OA. But this methodology can also later on be easily implemented for other conditions.

This platform not only supports efficient data processing but also fosters the translation of technological advancements into actionable clinical applications, ultimately contributing to a more personalized and precise approach to knee OA care and research.

### 5.1. Data Collection Standardization

To ensure high-quality data, it is essential to develop a standardized approach for gathering data from diverse sources, ensuring uniformity and interoperability across all data categories. This requires the establishment of robust data structures, schemas, and metadata protocols that can accommodate self-reported evaluations, clinical assessments, and data from activity trackers.

The protocol presented above adheres to strict guidelines, ensuring the validity and reliability of the collected data. By applying these rigorous standards, the methodology guarantees consistency across different data collection methods and devices, enabling seamless integration and analysis. This uniform framework is critical for minimizing variability, reducing errors, and enhancing the overall reliability of multisource data aggregation.

Such standardization not only facilitates accurate data comparison but also supports comprehensive analyses that can translate into actionable insights for both research and clinical applications.

### 5.2. Data Integration and Quality Insurance

Effective data mapping and transformation techniques are critical in converting raw data from various sources into a standardized format [57]. This process involves establishing detailed data mapping rules, data cleaning, normalization, aggregation, and enrichment to ensure data integrity, accuracy, and completeness [58]. Additionally, metadata management procedures are implemented to document and monitor metadata associated with connected datasets, supporting data governance, lineage tracking, and impact analysis [59].

### 5.3. Data Storage and Synchronization

A cloud-based storage service, such as Google Cloud Storage, is utilized to store integrated data, ensuring data consistency, availability, and durability. Scalable and robust storage technologies and synchronization techniques are employed to facilitate the capture, storage, and syncing of varied data from different sources [60]. This enables researchers and clinicians to extract actionable insights and make informed clinical decisions using integrated datasets.

### 5.4. Analytical Approaches

A range of statistical methods and analytical tools are employed to identify optimal combinations that yield maximum clinical insights from the dataset. Exploratory data analysis is performed to obtain an initial understanding of combined datasets, recognize trends, patterns, and outliers, and develop hypotheses for further research [61]. Descriptive statistics and data visualization techniques, such as interactive dashboards, are used to visually represent data patterns, trends, and anomalies.

### 5.5. Advanced Analytics and Artificial Intelligence

Statistical modeling approaches, including regressions, time series analysis, and multivariate analysis, are used to discover correlations and associations in the combined datasets. Machine learning algorithms are applied to create predictive models, categorize data, and derive insights from combined information [62]. Various models, including supervised, unsupervised, and reinforcement learning methods, are tested and validated to uncover concealed patterns and correlations among combined data [63].

### 5.6. Feedback Mechanisms and Iterative Improvement

Feedback mechanisms are essential to continuously improve the system. An iterative strategy is employed, gathering feedback from stakeholders, including clinicians, researchers, and individuals with knee OA [64]. This input is used to refine data collection methods, integration processes, and analytical capabilities, ensuring the framework remains user-centered and effective in clinical environments [65].

### 5.7. Scalability and Flexibility

The framework is designed with modular components and architectures that can easily be adjusted to evolving clinical requirements and support future expansions. The pipeline is composed of interchangeable modules, each with a distinct purpose, including data acquisition, processing, storage, or analysis. This modular design enhances flexibility, extensibility, and reusability, enabling easy addition of new functionalities or modification of current ones with minimal disturbance.

By adopting this comprehensive approach, the framework can effectively collect, integrate, and analyze data from diverse sources to gain insights into the impact of OA and its effects on individual needs and behaviors.

## 6. Pilot Data Processing and Preliminary Results

To assess the preliminary feasibility of the proposed hybrid assessment framework and to obtain exploratory insights into the discriminative potential of the collected variables, a proof-of-concept pilot study was conducted with a limited sample (n = 40). Given the small sample size, all reported group differences should be interpreted as exploratory rather than confirmatory. These results are intended to illustrate the framework’s potential and inform future adequately powered studies, not to draw definitive conclusions about group differences. All participants completed the full supervised assessment battery described in Section 4, including questionnaires, clinical tests, and technology-supported evaluations, including unsupervised data from wearable sensors. The study protocol was approved by the Institutional Ethics Committee of the Institut National de Santé Publique (INSP)-Bujumbura-Burundi and registered under reference number CIE /08/2024. Participants with knee OA were recruited from the outpatient rheumatology clinic of the INSP, between September 2024 and January 2025. Healthy controls were recruited via community advertisements from the same geographic area. All participants provided informed consent. To ensure privacy, the data used in this study were deidentified before analysis. Participants did not receive any compensation for their involvement.

### 6.1. Data Integration Pipeline

Raw data from multiple sources (demographic information, questionnaire scores, clinical test results, and sensor outputs) were consolidated using a custom data processing script. The integration process began with importing each data sheet and handling missing values according to predefined rules; for example, for the WOMAC questionnaire, missing responses were imputed using the average of available items when permitted by the scoring guidelines. For bilaterally measured parameters such as strength, range of motion, and gait metrics, the mean of the left and right sides was calculated to obtain composite variables, thereby reducing dimensionality and improving stability. All data frames were then merged by participant identifier to create a single master dataset.

From this dataset, participants with bilateral knee OA and healthy controls were selected. All continuous variables were standardized to z-scores (mean = 0, standard deviation = 1) to allow direct comparison across variables measured on different scales.

A heatmap was then generated from the normalized data matrix, where rows represented participants and columns represented variables. Rows were ordered by first grouping controls and patients separately, performing hierarchical clustering within each group, and then concatenating the two clusters. Column clustering was applied to identify groups of variables that co-vary. In addition, to quantify the association of each variable, Pearson correlation coefficients between each continuous variable and this group variable were computed.

A total of 40 participants were enrolled, comprising 20 patients with bilateral knee osteoarthritis (OA) and 20 age-matched healthy controls (Table 2). The OA group had a higher proportion of females (75% vs. 40%, *p* = 0.025) and a significantly higher body mass index (BMI: 29.15 ± 5.21 vs. 24.74 ± 5.07 kg/m^2^, *p* = 0.010). Height was slightly lower in patients (1.60 m vs. 1.65 m, *p* = 0.018), while weight did not differ significantly (77.40 vs. 68.60 kg, *p* = 0.074). Age was comparable between groups (57.85 vs. 52.65 years, *p* = 0.067). Given the exploratory nature of this pilot study, these between-group differences (particularly in sex distribution and BMI) were not controlled for but are reported to inform interpretation of subsequent outcome comparisons.

### 6.2. Heatmap Analysis

**Figure 2:**
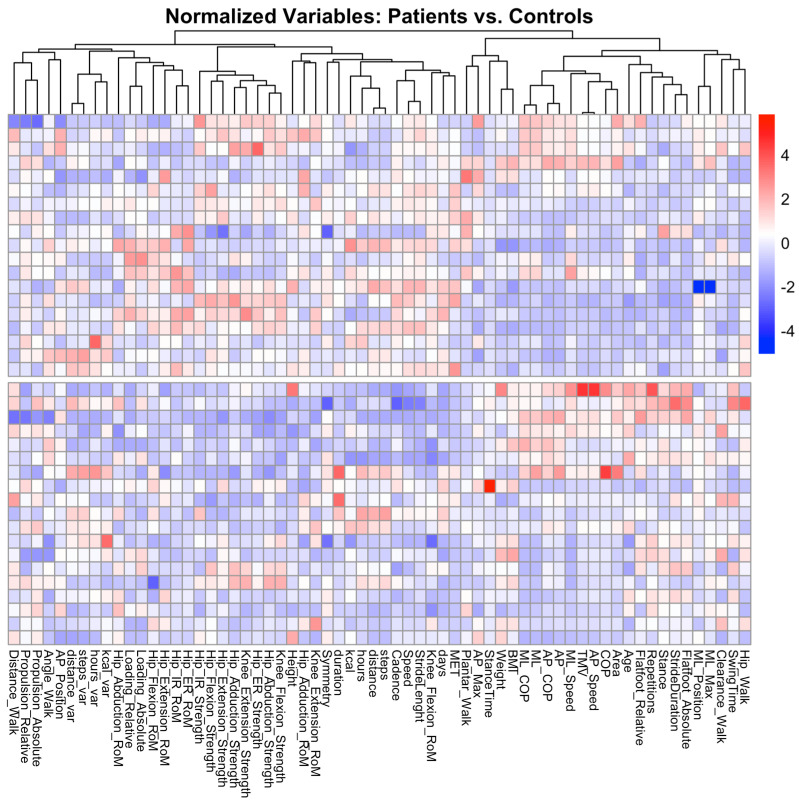
Heatmap summarizing normalized results for patients (**top**) and control (**bottom**).

The resulting heatmap (Figure 2) shows the 40 participants and all continuous variables after normalization, with blue indicating values below the overall mean and red values above the mean. A clear visual separation between the two groups is evident: most variables show systematically higher or lower values in patients compared to controls. Within the patient group, two sub-clusters are visible, suggesting possible heterogeneity in disease severity or functional compensation strategies. Several variables cluster together, indicating strong inter-correlations, for example, gait speed, stride length, and 6 min walk test distance. The heatmap confirms that the multidimensional assessment captures a broad range of impairments and activity limitations that collectively distinguish OA patients from healthy controls.

The visual separation observed in Figure 2 is encouraging for the framework’s discriminative potential; however, due to the small sample size, this pattern requires validation in larger, independent cohorts. The identified sub-clusters within the patient group may reflect heterogeneity in disease severity, but could also represent random variation given the limited number of participants.

### 6.3. Correlations

**Figure 3:**
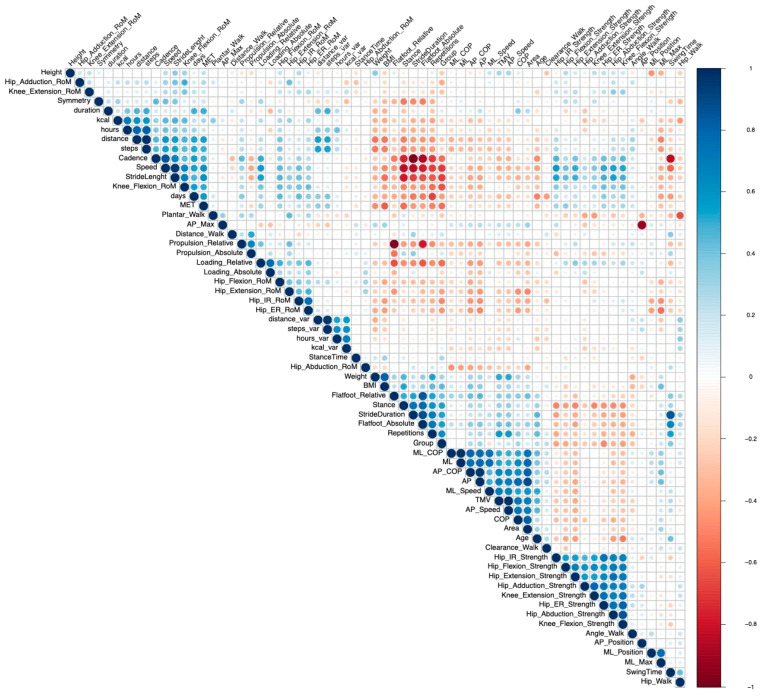
Correlogram showing the association between the different collected data.

Positive correlations (higher values in patients) were observed for several clinical and functional parameters. The highest positive coefficient was found for stance phase duration (r = 0.537), indicating that patients spend a longer proportion of the gait cycle in stance compared to controls. Flatfoot relative phase (r = 0.413) also showed moderate positive correlations, reflecting increased foot flat contact. Additionally, five-times sit-to-stand time (r = −0.435) was negatively correlated with group, and several balance-related variables such as AP_COP (r = 0.353), TMV (r = 0.463), and area (r = 0.302) were higher in patients, indicating worse postural control. All correlations are plotted in Figure 3.

Negative correlations (lower values in patients) were more numerous and generally stronger. The most discriminative negative correlations included gait speed (r = −0.698), stride length (r = −0.680), cadence (r = −0.552), and loading relative phase (r = −0.570), all indicating slower, shorter, and less propulsive walking patterns in patients. Knee flexion strength (r = −0.526) and hip flexion strength (r = −0.393) were also lower in patients, as were several range of motion measures (e.g., knee flexion RoM, r = −0.412; hip flexion RoM, r = −0.396).

These preliminary findings suggest that objective sensor-based measures may be effective at distinguishing individuals with knee OA from healthy controls. However, the correlation coefficients reported (ranging from −0.70 to 0.54) are based on a small sample and may be unstable; confidence intervals are wide and should be interpreted with caution. Future studies with larger samples are needed to obtain stable effect size estimates and to determine which variables most robustly discriminate between groups. The correlation analysis provides a data-driven basis for selecting the most informative features for future machine learning models aimed at automated OA assessment and progression monitoring.

### 6.4. Practical Implications

The pilot data processing pipeline proved robust and reproducible. The heatmap and correlation plot not only validated the overall framework but also highlighted the most informative variables for future analyses. The integration of unsupervised sensor data, although available for only a subset of participants, already shows promise for capturing real-world physical activity patterns. In the full study, the same pipeline will be applied to a larger sample, and the results will be used to train predictive models for disease severity, progression, and response to intervention.

## 7. Challenges

While the proposed innovative method is seen as a potential opportunity to revolutionize clinical assessment in patients with knee OA, various challenges need to be acknowledged, and in return, be addressed to fully leverage this methodology. These challenges can be reported through five distinct levels, such as the participants, the technology, the clinicians, the data, and the policymaker levels.

### 7.1. Participant-Related Challenges

Individuals with knee OA share common characteristics related to their age, beliefs about new technologies, digital literacy, as well as language and cultural issues [19,66,67]. These participants’ traits pose significant challenges in the technology implementation process [19,66]. In fact, most individuals with OA are of the older generation who face serious difficulties in using advanced technologies due to the lack of digital literacy, along with user interface complexity that can discourage patient engagement or lead to errors in usage [67].

Furthermore, patients’ beliefs about sharing data online, and worries about data privacy and security pose significant challenges, which are exacerbated by the fact that some of older patients may undervalue longitudinal technological assessments, particularly if the traditional method is the only method they have experienced and have had a good experience with it [67,68]. Therefore, they do not have any meaningful reason to be assessed using this new approach. Additionally, in some contexts (countries), most of people speak local languages, while technologies are set up with languages that are not usually spoken in many countries, particularly in low- and middle-income countries [66,69]. Again, in some societies, where collecting data with technologies is seen as taboo, especially for women when technology is attached to private body parts like thighs, cultural issues may pose a significant challenge [66]. This is especially evident in Muslim societies where women are nearly fully clothed.

Moreover, while activity trackers offer valuable insights into different physical activity profiles, participants may encounter discomfort or feel imposed upon when wearing such devices. This may negatively impact their ability to continuously wear a smartwatch and/or an activity tracker (i.e., Fibion) for a prolonged period, which is normally required for data accuracy, as data collection lasts at least a week.

Clinicians should be aware of these challenges and be equipped with individually adapted strategies to overcome them throughout the assessment process, taking into account different contextual realities.

### 7.2. Technology-Related Challenges

Technology-related challenges can be categorized into the following three levels: affordability, accessibility, and user interface complexity [19,69]. In fact, on the one hand, advanced technologies are excessively expensive for both healthcare institutions and patients, consisting a well-known barrier to a widespread implementation. On the other hand, there are infrastructure gaps in terms of the lack of internet connectivity coverage, and power supply, particularly in remote or underdeveloped areas [19]. This may constitute a considerable limitation to a sustainable implementation, since most technologies are dependent on internet connection and power supply. Furthermore, the complexity of the user interface of new technologies can discourage not only patients’ engagement but also professional adoption. Finally, in some contextual realities, the long-term technical maintenance may suffer from the lack of specialist technicians to repair these technologies in case of malfunctions or breakdown. Indeed, these new technologies are newly introduced in a healthcare system where there are no technicians prepared for their maintenance.

### 7.3. Clinician-Related Challenges

Integrating multisensor assessment into existing daily clinical practice may pose challenges [69]. This can be mainly due to the lack of digital literacy among clinicians, resulting in resistance to adopting new technologies, or due to distrust about their effectiveness, and therefore slowing the full implementation of this new approach [19,67]. Addressing this issue requires clinician digital education and training on the use of new technologies and the interpretation of data from them, along with technical support on continuous data maintenance and technical troubleshooting.

The proposed multisensor framework requires substantial financial investment. Approximate device costs are presented in Table 3.

For routine clinical care, these costs may be prohibitive, particularly in low-resource settings. However, for specialized rehabilitation centers or research applications, the investment may be justified by the depth of data obtained.

For successful clinical implementation, we propose a tiered approach:

- Tier 1 (essential, ~$500): Questionnaires (VAS, WOMAC), 6MWT with step counter, single activity tracker. Provides 70–80% of relevant information.
- Tier 2 (specialized care, ~$1500): Add dynamometry and detailed activity monitoring (Fibion).
- Tier 3 (research only, $7000): Full framework including force plate and electronic goniometer.

We also propose device reduction strategies: Force plate can be replaced by clinical balance tests (Berg Balance Scale); Fibion can be replaced by smartphone-based tracking; Digisoles can be replaced by instrumented walkway or simple stopwatch. The choice should be guided by the clinical question, resources, and patient digital literacy.

### 7.4. Data-Related Challenges

One significant challenge is balancing the advantages of using big data with the drawbacks of potential data quality issues. While big data offer vast amounts of information, they frequently lack the precision and cleanliness essential for accurate analysis [70]. On the other hand, while clean data ensure both accuracy and reliability, they might be limited in scope, hindering the study’s findings [20]. In addition, integrating data from different sources requires careful methodology, which can be challenging due to differences in data formats and structures [20]. The lack of synchronization can present inconsistencies in the way findings are analyzed. Furthermore, we should take into consideration the risk of overfitting or underfitting models when integrating data, which could potentially lead to misrepresentative conclusions [71].

### 7.5. Policymaker-Related Challenges

As the adoption of a new way of practicing can successfully be achieved if policymakers are committed, numerous challenges at this level, such as resistance to change, and lack of stakeholders’ engagement, must be mentioned [68,69]. Resistance to change can occur to policymakers who are comfortable with traditional methods of assessment and can therefore resist adopting innovative approaches in favor of conservative ones [68]. This can also be influenced by the manufacturers of traditional method tools discouraging policies supporting innovative technologies.

The lack of policymaker engagement can also be explained by the lack of involving key stakeholders like patients, clinicians, and technicians in decision-making, along with inconsistent communication, creating confusion and poor policy reception, ultimately limiting the adoption of new technologies [19,68]. The lack of long-term vision on technology sustainability and scalability, which focuses only on the immediate costs over long-term gains, failing to provide frameworks for training healthcare professionals, and neglecting patient education on the use of new technologies, constitutes a considerable limitation [67,68].

## 8. Discussion

This study introduces an innovative hybrid methodology for patient assessment, combining cross-sectional data collection in supervised settings with continuous data collection in unsupervised modes. The primary goal of this approach is to gain a comprehensive understanding of impairments, activity limitation, and quality of life in patients with knee OA.

The proposed hybrid assessment integrates both supervised and unsupervised measurement methods, utilizing a diverse range of techniques such as self-reported data, clinical assessments, and continuous monitoring via wearable activity trackers. This multidisciplinary approach holds significant promise for advancing our understanding of knee OA. However, we acknowledge the challenges clinicians and researchers may face when implementing this hybrid model, particularly when working with older populations and adapting to new technologies.

### 8.1. Advantages of Unsupervised Assessment

Unsupervised measurement offers several transformative benefits. In fact, it enables remote data collection, providing insights into patients’ real-world activities in their ecological environments, and incorporates health literacy generation and integration, empowering patients to actively participate in managing their own health conditions [72]. This paradigm shift redefines the traditional relationship between patients and healthcare professionals, fostering a more collaborative and participatory model of care.

This approach’s flexibility—allowing data to be collected anytime and anywhere—makes it a valuable supplement to traditional supervised assessments. It can also serve as a viable alternative when supervised assessments are not feasible due to geographic, temporal, or healthcare resource constraints. Furthermore, unsupervised measurement reduces the workload on healthcare professionals, enhances the ubiquity of assessment and care, and provides decision-supporting information for all healthcare providers involved [72].

Given that current healthcare services often fail to address the specific needs of older adults with knee OA—who frequently face additional comorbidities—this holistic assessment approach could lead to significant improvements in clinical data collection and patient care. Prior research supports the potential of this approach to enhance clinical understanding and outcomes in this population [9].

### 8.2. Mutlisensor Assessment in Knee OA

The integration of multisensor assessments in knee OA care is particularly advantageous when combining cross-sectional and longitudinal evaluations. This strategy deepens our understanding of the physical activity limitations experienced by patients in their ecological environments. By longitudinally assessing patients, we gain insights into how activity limitations evolve over time, enabling clinicians to tailor and adapt interventions more effectively to the individual needs of knee OA patients.

This comprehensive approach bridges existing gaps in the understanding of OA’s clinical presentation and its implications for daily life. By evaluating patients over time, it becomes possible to identify patterns and trends in mobility and physical activity, facilitating more precise and individualized care plans. The added value of this methodology lies in its multisensory component, which addresses the limitations of traditional assessments and offers a promising solution for personalized, patient-centered care.

This holistic approach aligns closely with the biopsychosocial model in rehabilitation sciences, emphasizing person-centered care and promoting patient involvement in the awareness and management of their conditions [73]. It is imperative for healthcare professionals and policymakers to adopt this methodology to better understand the personalized needs of patients and design appropriate interventions. The technical complexity of implementing such evaluations requires ongoing training and collaboration between researchers, clinicians, and engineers [20].

### 8.3. Challenges and Ethical Considerations

Despite its potential, the adoption of this approach poses several challenges. Patient concerns about privacy and data security are significant barriers, particularly among older adults who may be wary of sharing personal data online. Without proper education about the security measures in place, such concerns could undermine trust and limit the adoption of multisensor assessments. Implementing secure authentification methods, ensuring patients have control over their data, and building trust through transparency are critical to overcoming these barriers. Ethical considerations, including consent, confidentiality, and autonomy, must be prioritized, particularly in home settings where evaluations are conducted [72].

The risk of data breaches and the potential for compromised patient history highlight the need for robust ethical guidelines and stringent security protocols. Additionally, resistance to change among policymakers, clinicians, and patients, especially those unfamiliar with technological assessments, may hinder implementation. Some older patients may undervalue longitudinal technological assessments, particularly if they have only experienced traditional methods.

Practical constraints, including financial limitations, lack of internet coverage, power supply issues, and low digital literacy, further complicate implementation. Moreover, the technical complexity of these evaluations necessitates ongoing training and collaboration between clinicians, researchers, and engineers to ensure successful adoption and sustained usage [72].

### 8.4. Limitations of the Pilot Validation

The primary limitation of this pilot study is the small sample size (n = 20 per group), which precludes definitive statements about group differences and limits the generalizability of the findings. The reported p-values and effect sizes should be considered exploratory; some associations may be spurious due to type I error, while clinically meaningful differences may have been missed due to limited power (type II error). The lack of an independent validation cohort further restricts the generalizability of the heatmap and correlation findings. These results serve as proof-of-concept to demonstrate the framework’s feasibility and data integration capabilities, not as definitive evidence of specific sensor-derived biomarkers for knee OA. Rigorous, adequately powered validation studies are required before any clinical implementation.

### 8.5. Illustrative Clinical Applications

Based on the patterns observed in this pilot study, preliminary decision thresholds are proposed in Table 4. These rules are hypothesis-generating and require prospective validation.

## 9. Conclusions

This perspective presents an AI-enabled multisensor assessment framework that bridges conventional clinical evaluations with continuous, real-world digital phenotyping to support smart and precision healthcare for individuals with knee OA. By integrating advanced tools and approaches, it addresses the limitations of traditional methods while leveraging the benefits of modern technology.

Despite the potential challenges discussed, the insights gained from this approach could significantly inform the targeted hip and knee OA population, healthcare professionals, and policymakers at different levels of decision-making. These insights would enable the effective assessment and management of knee OA, which remains one of the most disabling conditions among older adults. Integrating this new assessment methodology into clinical practice has the potential to personalize strategies for promoting an active lifestyle and improving mobility among older adults with knee OA.

Beyond its clinical utility, this methodology contributes to the broader fields of public health and data science, offering actionable insights for addressing societal challenges such as population aging and chronic disease management. By promoting active aging and enhancing the well-being of older adults, the approach aligns with public health goals focused on healthy and active aging.

Conducting such multidimensional and multisensor assessments is essential for obtaining a comprehensive understanding of the physical status of older adults. This holistic view is key to designing interventions that address individual needs, reduce disability, and improve quality of life for this vulnerable population.

## Figures and Tables

**Table 1:** Description and psychometric properties of the different tests included in the proposed multidimensional assessment.

Category	Name	Variables of Interest	Administration Duration	Interpretation	MDC	ICC (Intra-Rater)		ICC (Inter-Rater)
Supervised Assessment								
Questionnaires	VAS	Pain intensity	2 min	The higher the score, the worse the pain.	2 pts [33]	0.82–0.95 [33]		0.97 [34]
	WOMAC	Pain Stiffness Physical Function	12–15 min [35]	Higher score indicates more severe impairments [35,36].	3.30 pts [37]	0.989 [37]		-
	SF-20	Health status	5–7 min [38]	Higher scores indicate better health status [38].		0.96 [38]		-
	Tampa Scale for kinesiophobia	Level of pain-related fear of movement	About 10 min [39]	Higher scores indicate greater kinesiophobia [40].	3.9–8.9 pts [41]	0.73–0.99 [41]		
	SF-20	Health status	5–7 min [38]	Higher scores indicate better health status [38].		0.96 [38]		-
	GPAQ	Self-reported physical activity	10 min	-	-	0.37–0.94 [42]		
Clinical and functional evaluation	Goniometer	Passive ROM	10 min	Lower values indicate hypomobility.	-	-		-
	Dynamometer	Isometric hip and knee muscle strength	5 min	Lower values indicate muscle weakness.	-	Hip	0.95–0.97 [43]	0.95–0.98 [43,44]
						Knee	0.95 [45]	0.94 [46]
	6MWT	Number of steps, Distance, Cadence, Speed, Step symmetry	6 min	A higher score of 6MWT signifies better walking performance.	-	0.94–0.96 [47]		-
	Digit insoles			A higher value and step symmetry signifies positive outcome.	-	-		0.313–0.990 [48]
	5-times sit-to-stand test	Time taken to complete the 5-times sit-to-stand up test [49]	~1 min	The longer the time taken to complete the 5-times sit-to-stand test, the poorer the physical performance.	1.54 s [50]	0.94 [50]		0.99 [50]
	Balance (K-plates)	Ground reaction force, postural stability, Weight-bearing symmetry	~6 min	The smaller elliptical area and the center of pressure (COP) path length, the better the postural balance [51]. The larger the COP amplitude, the poorer the postural stability [52]	-	0.628 [53]		-
Unsupervised Assessment								
Remote monitoring	Fibion [51,54]	Sitting, walking and standing duration, light, moderate and vigorous physical activity (PA) duration, energy expenditure	At least 24 h	Greater score of total duration of PA and total energy expenditure signifies positive outcome.	-	Duration of activity in sitting (0.189), standing (0.459) and walking (0.227). Energy expenditure in sitting (0.806), standing (0.687) and walking (0.782) [51].		Duration of activity in sitting (0.87), standing (0.84) and walking (0.97) [55]. Total duration of physical activity (0.638) and 12 h total energy expenditure (0.743) [51].
	Polar watch [56]	Step number, distance walked, duration of PA, energy expenditure	7 days	Greater score signifies positive outcome.	-	-		-

**Table 2:** Exploratory group comparisons of outcome measures.

Category	Variable	Control N = 20 ^1^	Patient N = 20 ^1^	*p*-Value ^2^
Gait	Symmetry	97.50 [3.00]	97.50 [5.25]	0.64
	Cadence, step/m	120.45 (7.12)	106.70 (12.32)	<0.001
	Speed, m/s	5.90 (0.77)	4.32 (0.83)	<0.001
	Stance	59.68 [2.60]	62.10 [2.41]	<0.001
	Stride Duration, ms	998.90 (61.38)	1138.98 (140.29)	<0.001
	Stride Length, m	1.62 (0.16)	1.34 (0.14)	<0.001
	Swing Time, ms	400.53 (18.83)	427.13 (37.89)	0.009
	Stance Time, ms	590.75 [38.38]	698.25 [131.50]	<0.001
	Propulsion (relative)	38.00 [5.75]	32.50 [7.88]	0.028
	Flatfoot (relative)	51.00 [7.88]	58.00 [8.00]	0.003
	Loading (relative)	11.50 [2.13]	9.00 [2.00]	<0.001
	Propulsion (absolute)	219.63 (31.96)	233.25 (38.85)	0.23
	Flatfoot (absolute)	306.00 [51.25]	392.50 [108.50]	<0.001
	Loading (absolute)	68.75 [11.50]	63.25 [21.00]	0.26
Strength	Hip Abduction, kg	11.62 (2.30)	9.78 (2.55)	0.021
	Hip Adduction, kg	10.08 [4.63]	8.13 [2.86]	0.056
	Hip Flexion, kg	22.04 (6.89)	16.62 (5.59)	0.010
	Hip Extension, kg	17.59 (4.53)	15.00 (3.34)	0.047
	Hip internal rotation, kg	10.78 [5.78]	7.85 [4.89]	0.007
	Hip external rotation, kg	10.28 [4.08]	7.13 [2.63]	0.004
	Knee Flexion, kg	11.00 [4.93]	7.95 [2.41]	0.003
	Knee Extension, kg	16.48 [5.84]	12.73 [3.10]	0.024
Sit-to-Stand	Time (5 repetitions), s	10.50 [3.25]	20.00 [15.50]	<0.001
Amplitude	Hip Abduction, °	41.84 (15.60)	45.03 (16.59)	0.53
	Hip Adduction, °	37.05 (13.14)	27.25 (11.51)	0.017
	Hip Flexion, °	98.23 (20.07)	86.53 (21.89)	0.086
	Hip Extension, °	38.08 (8.53)	30.91 (7.43)	0.007
	Hip internal rotation, °	53.40 [46.55]	43.50 [10.05]	0.003
	Hip external rotation, °	46.00 [38.23]	44.25 [10.08]	0.050
	Knee Flexion, °	130.07 (11.46)	97.77 (26.62)	<0.001
	Knee Extension, °	17.01 (4.92)	14.28 (6.09)	0.13
Balance	COP, mm	60.10 [52.00]	74.80 [29.44]	0.022
	AP, mm	11.81 [11.81]	17.93 [8.93]	0.022
	ML, mm	3.06 [3.16]	2.98 [3.38]	0.71
	TMV, mm/s	6.61 [3.48]	7.66 [2.97]	0.064
	Area, mm^2^	15.82 [28.21]	32.10 [54.03]	0.056
	AP Speed, mm/s	6.19 [3.26]	7.52 [2.92]	0.036
	ML Speed, mm/s	1.46 [1.23]	1.32 [1.07]	0.26
Questionnaire	Duration, min/day	247.5 [225]	112.5 [337.5]	0.19
	MET	4890 [7240]	450 [1350]	<0.001
Activity	Distance, km per day	7.17 (3.21)	5.14 (3.62)	0.070
	Variability Distance	2.89 (1.33)	2.51 (1.66)	0.43
	Hours per day	4.87 (1.92)	4.64 (2.31)	0.74
	Variability Hours	1.69 [0.87]	1.54 [1.18]	0.44
	Kcal per day	2181.70 (365.13)	2001.22 (320.03)	0.10
	Variability Kcal	236.39 [168.75]	255.67 [179.00]	0.99
	Steps per day	10,333.21 [7062.18]	7223.64 [8233.86]	0.076
	Variability steps	4582.01 (2127.53)	3956.67 (2672.08)	0.42

^1^ Mean (SD); n (%); Median [IQR], ^2^ Welch two sample *t*-test; Pearson’s Chi-squared test; Wilcoxon rank sum test; Wilcoxon rank sum exact test.

**Table 3:** Cost of the equipment.

Device	Approximate Cost (USD)	Purpose
Kinvent K-Move (goniometer)	$750	ROM assessment
Kinvent K-Push (dynamometer)	$1250	Strength testing
Kinvent K-Plate (force plate)	$3000	Balance assessment
Digisoles (connected insoles)	$2000	Gait analysis
Polar M200 smartwatch	$100	Activity tracking
Fibion Sens	$100	Detailed ADL monitoring
**Total approximate cost**	**$7200**	Per clinical setup (excluding multiple patient devices)

**Table 4:** Preliminary clinical decision rules derived from the multisensor framework.

Clinical Question	Key Metrics	Decision Threshold	Suggested Intervention
Strengthening vs. gait retraining?	Step length vs. cadence discordance	Step length >20% below age norm with normal cadence	Gait retraining + graded exposure
True weakness vs. fear avoidance?	Dynamometry strength vs. real-world steps	Strength >10 kg but steps <4000/day	CBT + graded activity prescription
Surgical referral timing?	Step variability + strength asymmetry	Day-to-day step variability >50% AND strength asymmetry >30%	Consider total knee arthroplasty
Exercise intensity prescription?	Fatigue pattern from continuous monitoring	Step length decline >15% after 3 min of walking	Interval training (short bouts with rest)

Note: These thresholds are derived from exploratory pilot data (n = 20 per group) and should be validated in larger prospective cohorts before clinical implementation.

## Data Availability

The data are available upon request to the corresponding authors.

## Supplementary Materials

The following supporting information can be downloaded at: https://www.mdpi.com/article/10.3390/s26113563/s1, Supplementary Material S1: Description of the Assessment Tools Used. References [74,75,76,77,78,79,80,81,82,83,84,85,86,87,88,89,90,91,92] are cited in Supplementary Materials.
