# Supplementary material for: Wearable Sensors and Artificial Intelligence for Ecological Knee Osteoarthritis Assessment: Development and Feasibility of a Hybrid Digital Phenotyping Framework
Source: Sensors (Basel). 2026 Jun 3;26(11):3563. doi: 10.3390/s26113563 (PMC13259533; doi:10.3390/s26113563)
Supplement: Supplementary file 1 [file sensors-26-03563-s001.zip › sensors-4281062-supplementary.pdf]

Supplementary Material S1: Description of the Assessment Tools Used

Data collection was conducted based on the International Classification of Functioning, Disability and Health (ICF) framework (WHO 2001). Applying this model to knee OA facilitates the identification of impairments, activity limitations, and the associated impact on participants' quality of life [1, 2].

Phase 1- Conventional supervised clinical assessment

Patient-reported outcomes measures

Current pain intensity was assessed using the Visual Analogue Scale (VAS; ICC 0.82–0.95; MDC 2 pts), a self-reported 10 cm scale with verbal anchors from "no pain" to "the most intense pain imaginable [3-5]." Perceived physical functioning was assessed with the Western Ontario and McMaster Universities Arthritis Index (WOMAC; ICC 0.989; MDC 3.30 pts), a validated questionnaire assessing three components: pain (5 items, 0–20), stiffness (2 items, 0–8), and physical function (17 items, 0–68) [6]. Total scores are standardized as a percentage from 0 to 100 [7, 8]. Patients' fear of movement was assessed using the Tampa Scale for Kinesiophobia (TSK; ICC 0.73–0.99; MDC 3.9–8.9 pts), a 17-item self-reported scale with scores ranging from 17 to 68, where higher scores indicate greater kinesiophobia [9, 10]. Patients' perceived quality of life was investigated using the Short Form-20 (SF-20; ICC 0.96), a 6-domain questionnaire covering physical functioning, role functioning, social functioning, mental health, current health perceptions, and pain, with all scores converted to a 0–100 scale where higher scores indicate better functioning [11]. Self-reported physical activity was assessed with the Global Physical Activity Questionnaire (GPAQ; ICC 0.37–0.94), a 19-item instrument capturing activity across work, transport, and discretionary domains [12]. Energy expenditure was calculated using metabolic equivalents of task (MET), enabling categorization of physical activity levels into vigorous, moderate, and light intensity [12-14].

Functional performance tests

Activity limitation was assessed with the 6-Minute Walk Test (6MWT; ICC 0.94–0.96), performed in a 30-metre corridor [10]. The participant is instructed to walk at maximum constant speed for 6 minutes. Lower-limb functional performance was assessed using the Five Times Sit-to-Stand Test (5×STS; ICC 0.94; MDC 1.54 s), measuring the time taken to rise from a seated position and return to sitting five times as quickly as possible without using hands for support [15-17]. Completion time is recorded by an experienced physiotherapist using a stopwatch.

Phase 2 - Cross-Sectional Technology-Supported Assessment

Electronic Goniometry (Range of motion)

Passive hip and knee ROM was measured in standardized positions to minimize errors and movement compensations using the Kinvent® K-Move electronic goniometer, a valid and reliable tool for measuring joint mobility [18]. Three trials were performed per joint and per side, with the maximum value recorded. Hip assessments included flexion, extension, abduction, adduction, internal rotation, and external rotation; knee assessments include flexion and extension. This approach eliminates the observer bias inherent to traditional manual goniometry and enables long-term data storage for tracking disease progression.

Hand-Held Dynamometry (Muscle strength)

Isometric hip and knee muscle strength was assessed using the Kinvent® K-Push Bluetooth-connected hand-held dynamometer (hip ICC intra-rater 0.95–0.97, inter-rater 0.95–0.98; knee ICC intra-rater 0.95, inter-rater 0.94) [19]. Three maximal voluntary isometric contractions were performed per muscle group and per side, with the peak value recorded. Standardized instructions are given: "At the count of three, push as hard and as fast as you can and hold that contraction until I say relax [19, 20]." Muscle groups assessed include hip flexion, extension, abduction, adduction, internal and external rotation, and knee flexion and extension [21].

Force plate assessment

Static bipedal balance was assessed using the Kinvent® K-plate Bluetooth-connected force plate system following the modified Clinical Test of Sensory Interaction in Balance (mCTSIB) protocol (ICC intra-rater 0.628). Variables included centre-of-pressure (COP) path length, elliptical area, and anterior-posterior (AP) and mediolateral (ML) sway amplitude and speed [22]. The device connected via Bluetooth to a tablet or smartphone, allowing real-time data display and secure storage.

Smart Insole Gait Analysis

Participants performed the 6MWT while wearing Digisoles® connected smart insoles (inter-rater ICC 0.313–0.990) [23-27]. This augmented the standard 6MWT with real-time spatiotemporal gait parameters: cadence, walking speed, stride length, stride duration, stance and swing phase durations (absolute and relative), flatfoot phase (absolute and relative), loading phase (relative), propulsion phase (relative), gait symmetry, and performance fatigability [23-27]. This provided detailed and objective insights into walking performance that go beyond conventional clinical observation, enabling real-time analysis of gait patterns and their fluctuations over time, including fatigue-induced changes in stride length, cadence, or stability that would go unnoticed in a standard 6MWT.

Phase 3 — 7-day unsupervised home monitoring

The Polar® M200 smartwatch continuously tracked step count, distance walked, physical activity duration and intensity, and energy expenditure for up to 7 days [28]. Participants are instructed to wear the device continuously, removing it only while showering or during nighttime hours. Data were uploaded to the Polar application following the monitoring period. For practical reasons, heart rate monitoring via the chest belt was not used in this study.

The Fibion® Sens activity tracker provides a granular minute-by-minute profile of activities of daily living, including daily time spent in sitting, standing, and walking; energy expenditure; activity intensity categories (vigorous, moderate, light); sedentary behavior (sitting health risk points, 0 to −100); and exercise health benefit scores (0 to 100) [29-31]. Inter-rater ICC values for the Fibion device are: duration of activity in sitting (0.87), standing (0.84), and walking (0.97); total duration of physical activity (0.638); and 12-hour total energy expenditure (0.743) [29-31].

References

1. Kifley, A., et al., *Australian arm of the International Spinal Cord Injury (Aus-InSCI) Community Survey: 3. Drivers of quality of life in people with spinal cord injury.* Spinal Cord, 2023. **61**(3): p. 185-193.
2. McDougall, J., V. Wright, and P. Rosenbaum, *The ICF model of functioning and disability: incorporating quality of life and human development.* Dev Neurorehabil, 2010. **13**(3): p. 204-11.
3. Eliana, R., R. Carolina, and S. Yannely, *No. 377 Intrarater Reliability and Level of Agreement of the Visual Analogue Scale, Goniometry, Hand-Held Dynamometry and the Six-Minute Walk Test in Persons With Knee Osteoarthritis.* PM&R, 2014. **6**: p. S166-S166.
4. MacDowall, A., et al., *Validation of the visual analog scale in the cervical spine.* J Neurosurg Spine, 2018. **28**(3): p. 227-235.
5. Suksri, T., C. Gaogasigam, and S. Boonyong, *Intra-rater and inter-rater reliability and minimum detectable change of visual analog scale and digital goniometer in patients with subacute unilateral lateral ankle sprain.* Chulalongkorn Medical Journal, 2022. **66**(4): p. 411-417.
6. Bellamy, N., et al., *Validation study of WOMAC: a health status instrument for measuring clinically important patient relevant outcomes to antirheumatic drug therapy in patients with osteoarthritis of the hip or knee.* J Rheumatol, 1988. **15**(12): p. 1833-40.
7. Clement, N.D., et al., *What is the Minimum Clinically Important Difference for the WOMAC Index After TKA?* Clin Orthop Relat Res, 2018. **476**(10): p. 2005-2014.

8. Burch, F.X., et al., *Evaluating the benefits of patterned stimulation in the treatment of osteoarthritis of the knee: a multi-center, randomized, single-blind, controlled study with an independent masked evaluator.* Osteoarthritis Cartilage, 2008. **16**(8): p. 865-72.
9. Hidaka, R., et al., *Association of high kinesiophobia and pain catastrophizing with quality of life in severe hip osteoarthritis: a cross-sectional study.* BMC Musculoskelet Disord, 2023. **24**(1): p. 388.
10. Machado, S., et al., *Knee Osteoarthritis: Kinesiophobia and Isometric Strength of Quadriceps in Women.* Pain Res Manag, 2022. **2022**: p. 1466478.
11. Vlaeyen, J.W.S., et al., *Fear of movement/(re)injury in chronic low back pain and its relation to behavioral performance.* Pain, 1995. **62**(3): p. 363-372.
12. Bull, F.C., T.S. Maslin, and T. Armstrong, *Global physical activity questionnaire (GPAQ): nine country reliability and validity study.* J Phys Act Health, 2009. **6**(6): p. 790-804.
13. Keating, X.D., et al., *Reliability and Concurrent Validity of Global Physical Activity Questionnaire (GPAQ): A Systematic Review.* Int J Environ Res Public Health, 2019. **16**(21).
14. Committee, I.R., *Guidelines for data processing and analysis of the International Physical Activity Questionnaire (IPAQ)-short and long forms.* http://www. ipaq. ki. se/scoring. pdf, 2005.
15. Naili, J.E., et al., *The centre of mass trajectory is a sensitive and responsive measure of functional compensations in individuals with knee osteoarthritis performing the five times sit-to-stand test.* Gait Posture, 2018. **62**: p. 140-145.
16. Yenişehir, S., et al., *Reliability and validity of Five Times Sit to Stand Test in pregnancy-related pelvic girdle pain.* Musculoskelet Sci Pract, 2020. **48**: p. 102157.
17. Esbjörnsson, A.C. and J.E. Naili, *Functional movement compensations persist in individuals with hip osteoarthritis performing the five times sit-to-stand test 1 year after total hip arthroplasty.* J Orthop Surg Res, 2020. **15**(1): p. 151.
18. Tekin, F., T. Can-Akman, and A. Kitiş, *Evaluation of the validity and reliability of the KFORCE Sens® electrogoniometer in evaluation of wrist proprioception.* Hand Surg Rehabil, 2022. **41**(2): p. 183-188.
19. Mentiplay, B.F., et al., *Assessment of Lower Limb Muscle Strength and Power Using Hand-Held and Fixed Dynamometry: A Reliability and Validity Study.* PLoS One, 2015. **10**(10): p. e0140822.
20. Chopp-Hurley, J.N., et al., *Investigating the Test-Retest Reliability and Validity of Hand-Held Dynamometry for Measuring Knee Strength in Older Women with Knee Osteoarthritis.* Physiother Can, 2019. **71**(3): p. 231-238.
21. Hogrel, J., G. Ollivier, and C. Desnuelle, *Manual and quantitative muscle testing in neuromuscular disorders. How to assess the consistency of strength measurements in clinical trials?* Revue neurologique, 2006. **162**(4): p. 427-436.
22. Chen, B., et al., *Review of the Upright Balance Assessment Based on the Force Plate.* Int J Environ Res Public Health, 2021. **18**(5).
23. Chevance, G., et al., *Accuracy and Precision of Energy Expenditure, Heart Rate, and Steps Measured by Combined-Sensing Fitbits Against Reference Measures: Systematic Review and Meta-analysis.* JMIR Mhealth Uhealth, 2022. **10**(4): p. e35626.
24. St Fleur, R.G., et al., *Use of Fitbit Devices in Physical Activity Intervention Studies Across the Life Course: Narrative Review.* JMIR Mhealth Uhealth, 2021. **9**(5): p. e23411.
25. Aristizábal Pla, G., et al., *The Use of a Single Trunk-Mounted Accelerometer to Detect Changes in Center of Mass Motion Linked to Lower-Leg Overuse Injuries: A Prospective Study.* Sensors (Basel), 2021. **21**(21).
26. Trivedi, H.D. and E.B. Tapper, *Interventions to improve physical function and prevent adverse events in cirrhosis.* Gastroenterol Rep (Oxf), 2018. **6**(1): p. 13-20.
27. Schütte, K.H., et al., *Energy cost of running instability evaluated with wearable trunk accelerometry.* J Appl Physiol (1985), 2018. **124**(2): p. 462-472.

28. Flores, G., et al., *Heart rate variability activity in soccer athletes after a musculoskeletal injury*. J Rehabil Med, 2024. **56**: p. jrm24969.
29. Alkalih, H.Y., A.J. Pesola, and A. Arumugam, *A new accelerometer (Fibion) device provides valid sedentary and upright time measurements compared to the ActivPAL4 in healthy individuals*. Heliyon, 2022. **8**(10): p. e11103.
30. Yang, Y., et al., *Reliability and validity of a new accelerometer-based device for detecting physical activities and energy expenditure*. PeerJ, 2018. **6**: p. e5775.
31. Alsamman, R.A., et al., *Effect of night-time data on sedentary and upright time and energy expenditure measured with the Fibion accelerometer in Emirati women*. Diabetes Metab Syndr, 2022. **16**(2): p. 102415.
